# Supplementary material for: Evaluation of Hydroxyl Radical Reactivity by Thioether Group Proximity in Model Peptide Backbone: Methionine versus S-Methyl-Cysteine
Source: Int J Mol Sci. 2022 Jun 11;23(12):6550. doi: 10.3390/ijms23126550 (PMC9224496; doi:10.3390/ijms23126550)
Supplement: Supplementary file 1 [file ijms-23-06550-s001.zip › ijms-1748923-supplementary.pdf]

Supplementary Materials

Chryssostomos Chatgilialoglu *, Magdalena Grzelak , Konrad Skotnicki , Piotr Filipiak , Franciszek Kazmierczak , Gordon L. Hug , Krzysztof Bobrowski * and Bronislaw Marciniak *

* Correspondence: chrys@isof.cnr.it (CC); kris@ichtj.pl (KB); marcinia@amu.edu.pl; (BM)

Contents pages

Figures S1	S2
Figures S2	S3
Table S1	S4
Figure S3	S5
Figure S4	S6
Scheme S1	S7
Figure S5	S8-S11
Figure S6	S12
Table S2	S13
Figure S7	S14
Figure S8	S15
Scheme S2	S16
Figure S9	S17

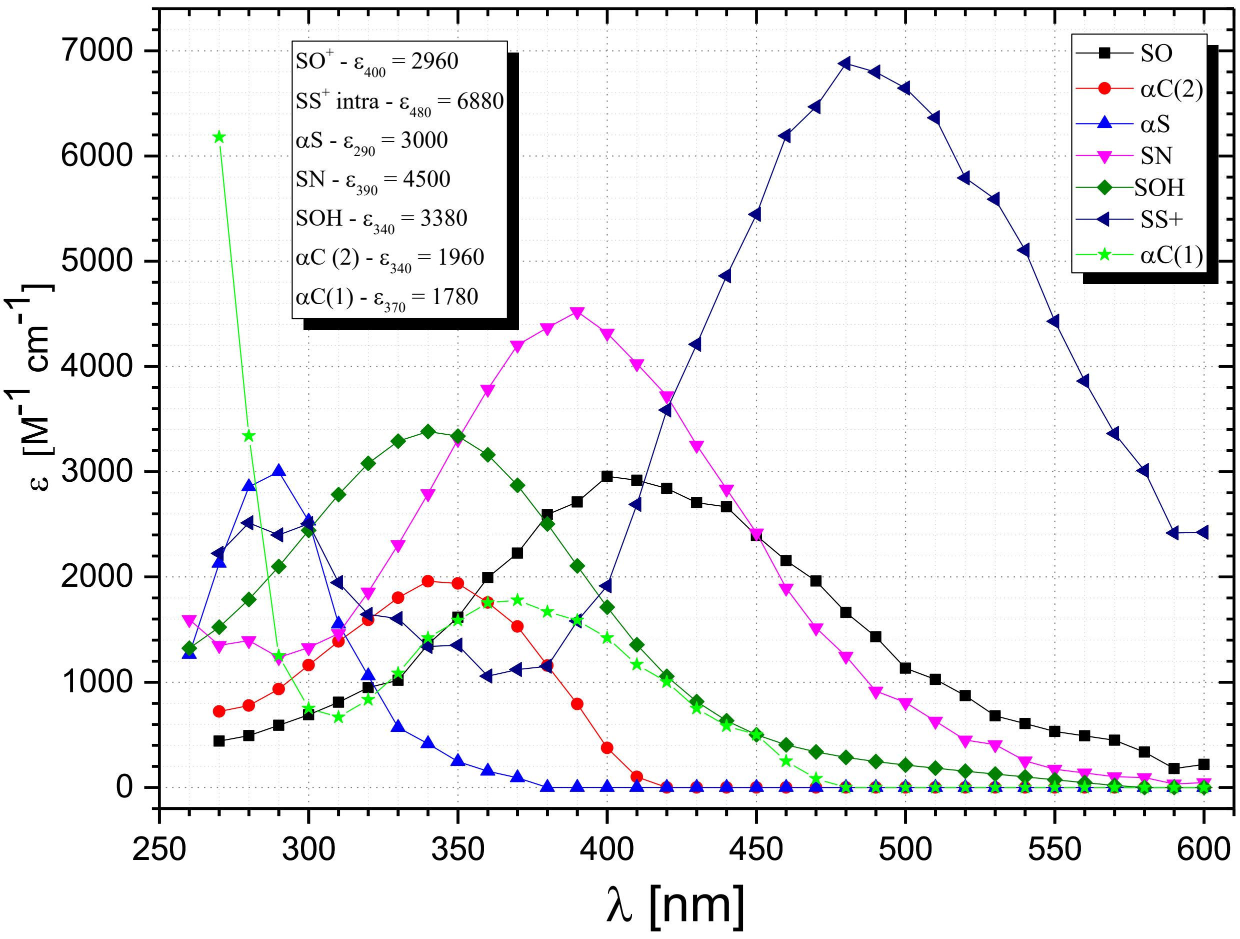

Figure S1. Reference spectra used in the resolution of the transient absorption spectra following •OH-induced oxidation of Met-containing derivative **1** and methylated Cys derivative **2**.

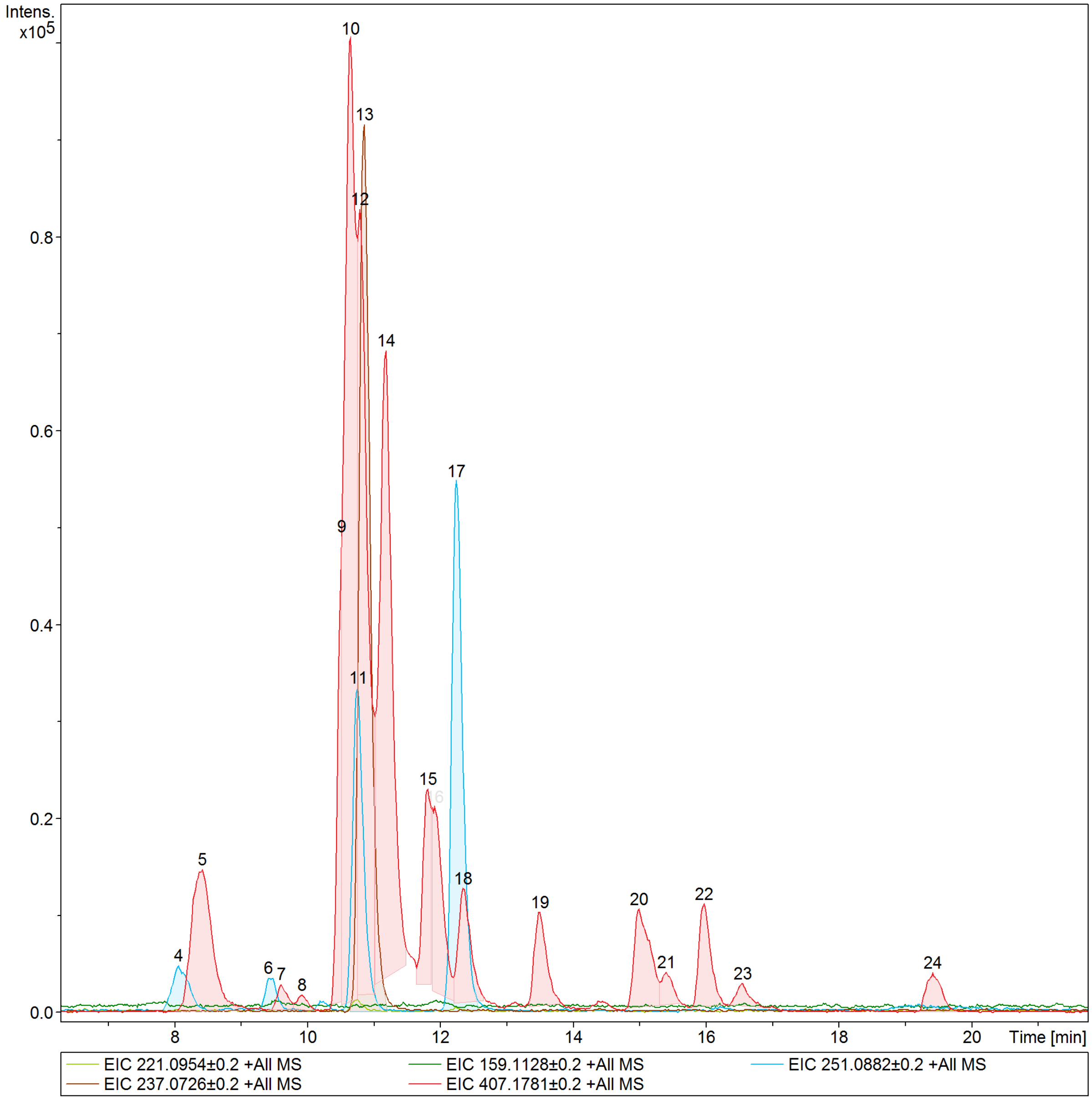

Figure S2. Fragments of LC-MS analysis for Met-containing derivative **1** after irradiation at 800 Gy at pH 7

Table S1. Conversion of Met derivative **1** (peak 3) after irradiation at 800 Gy at pH 7. The peaks 4-24 are shown in Figure S1.

Peak	RT [min] [b]	Peak Intensity [c]	MH^+, *m/z*
1	1.1	254430	221.0954
2	1.4	62161	159.1128
3 [a]	3.1	2954972	205.1005
4	8.1	4812	251.0882
5	8.4	14720	407.1781
6	9.4	3558	251.0882
7	9.6	2876	407.1781
8	9.9	1777	407.1781
9	10.5	49076	407.1781
10	10.7	100445	407.1781
11	10.8	33462	251.0882
12	10.8	82873	407.1781
13	10.9	91583	237.0726
14	11.2	68244	407.1781
15	11.8	23033	407.1781
16	11.9	21157	407.1781
17	12.2	54770	251.0882
18	12.4	12759	407.1781
19	13.5	10320	407.1781
20	15.0	10622	407.1781
21	15.4	4124	407.1781
22	16.0	11161	407.1781
23	16.6	2965	407.1781
24	19.4	4069	407.1781

[a] Starting material (Met derivative **1**); [b] retention time; [c] relative values.

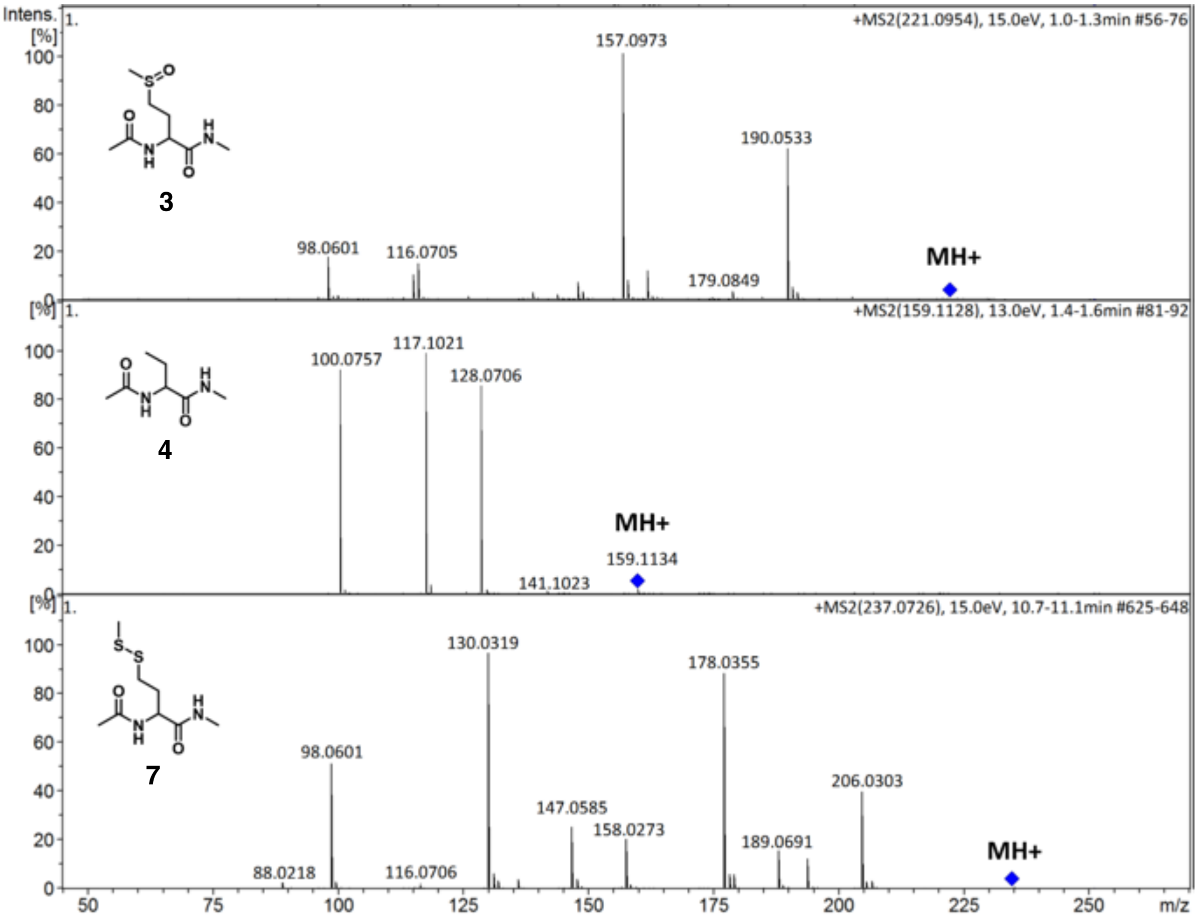

Figure S3. High-resolution MS/MS spectra of sulfoxide **3** (*m/z* 221.0954), α-aminobutyric derivative **4** (*m/z* 159.1134) and disulfide **7** (*m/z* 237.0726).

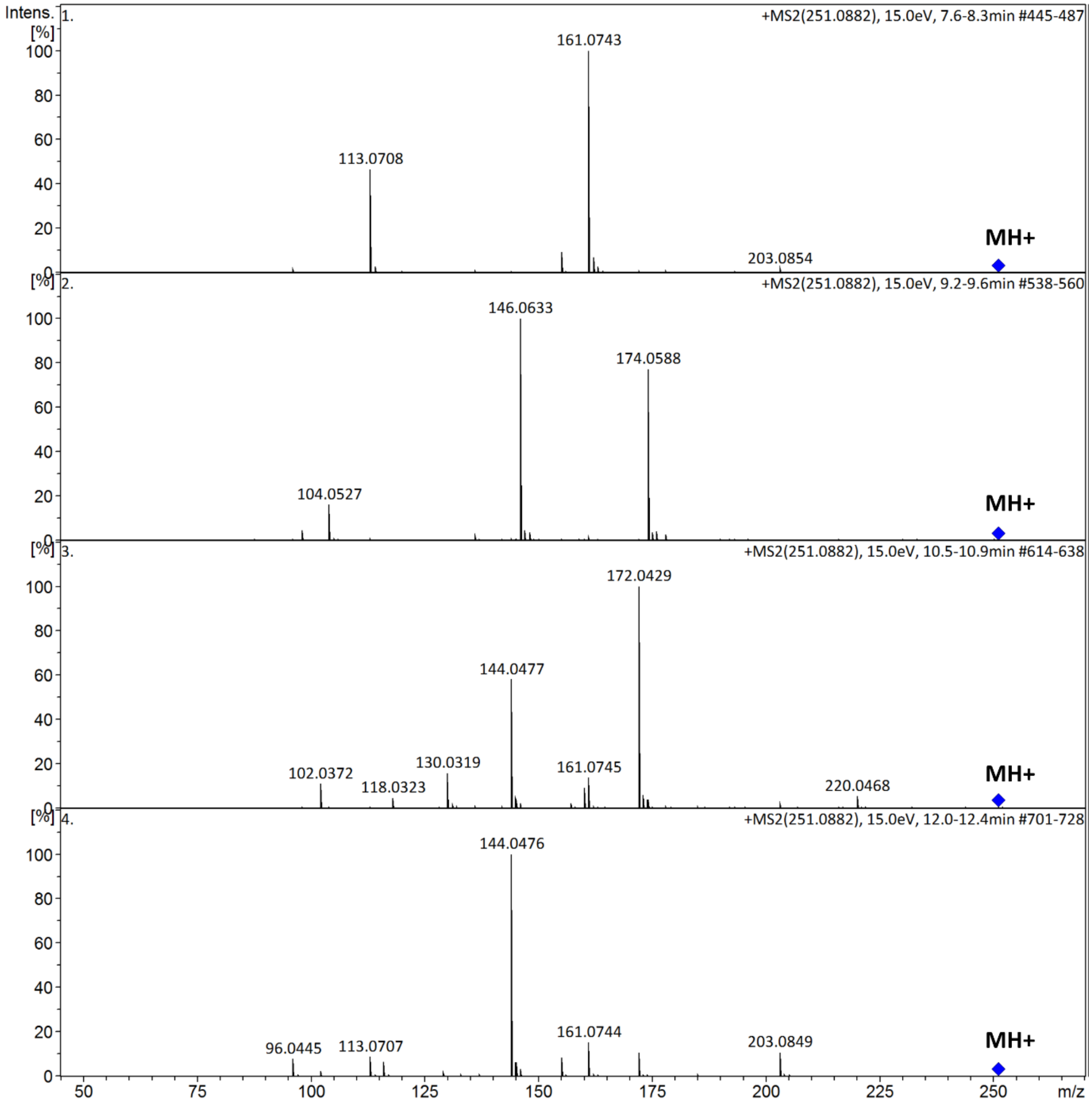

Figure S4. High-resolution MS/MS spectra of four products having *m/z* 251.0882 (see Table S1) assigned to the cross-termination of **αC(1)**$^{\bullet}$, **αC(2)**$^{\bullet}$, **αS(1)**$^{\bullet}$ and **αS(2)**$^{\bullet}$ with $CH_3S^{\bullet}$ (from the top to the bottom based on the analysis reported in Scheme S1).

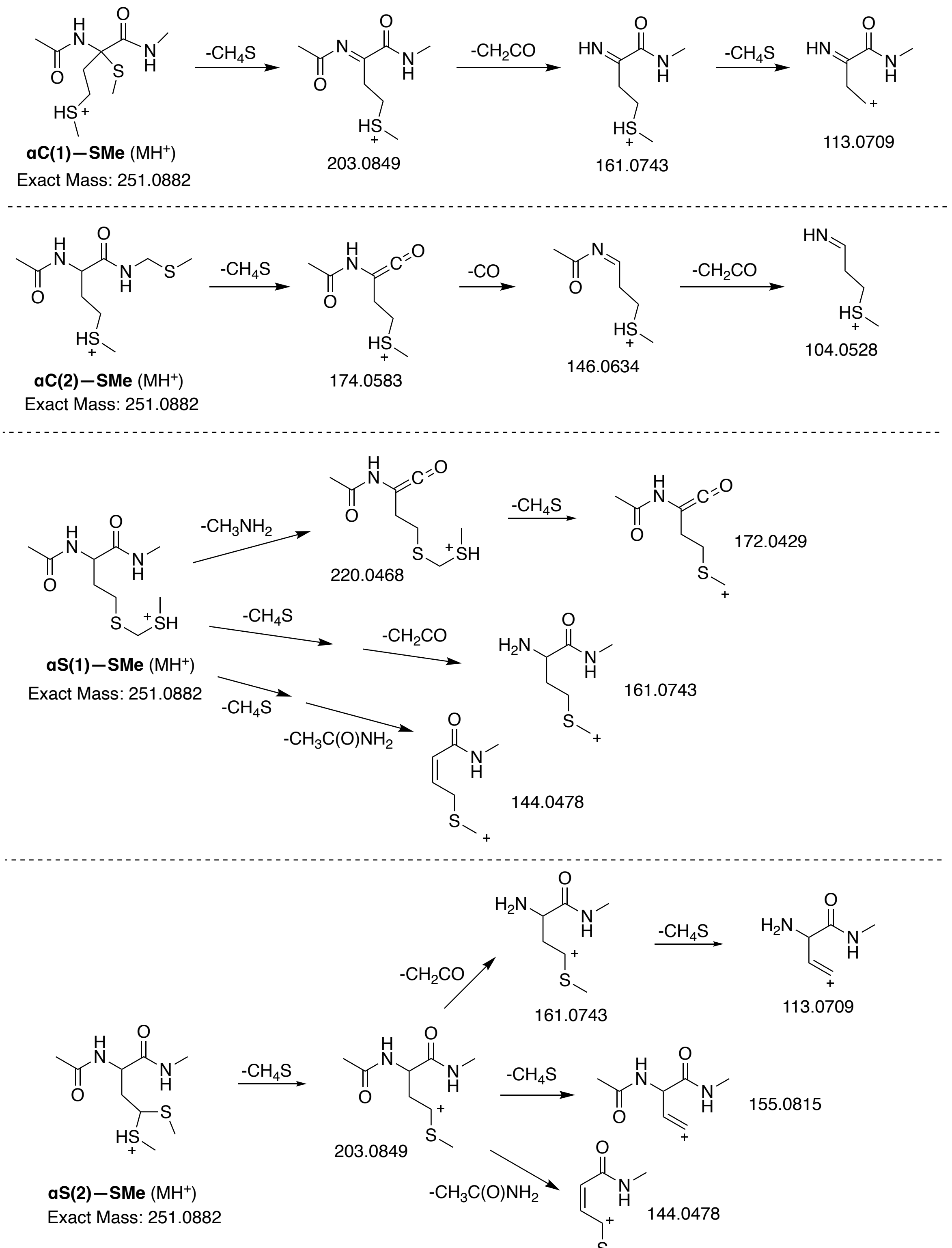

Scheme S1. Analysis of high-resolution MS/MS spectra of the four products having *m/z* 251.0882 reported in Figure S4.

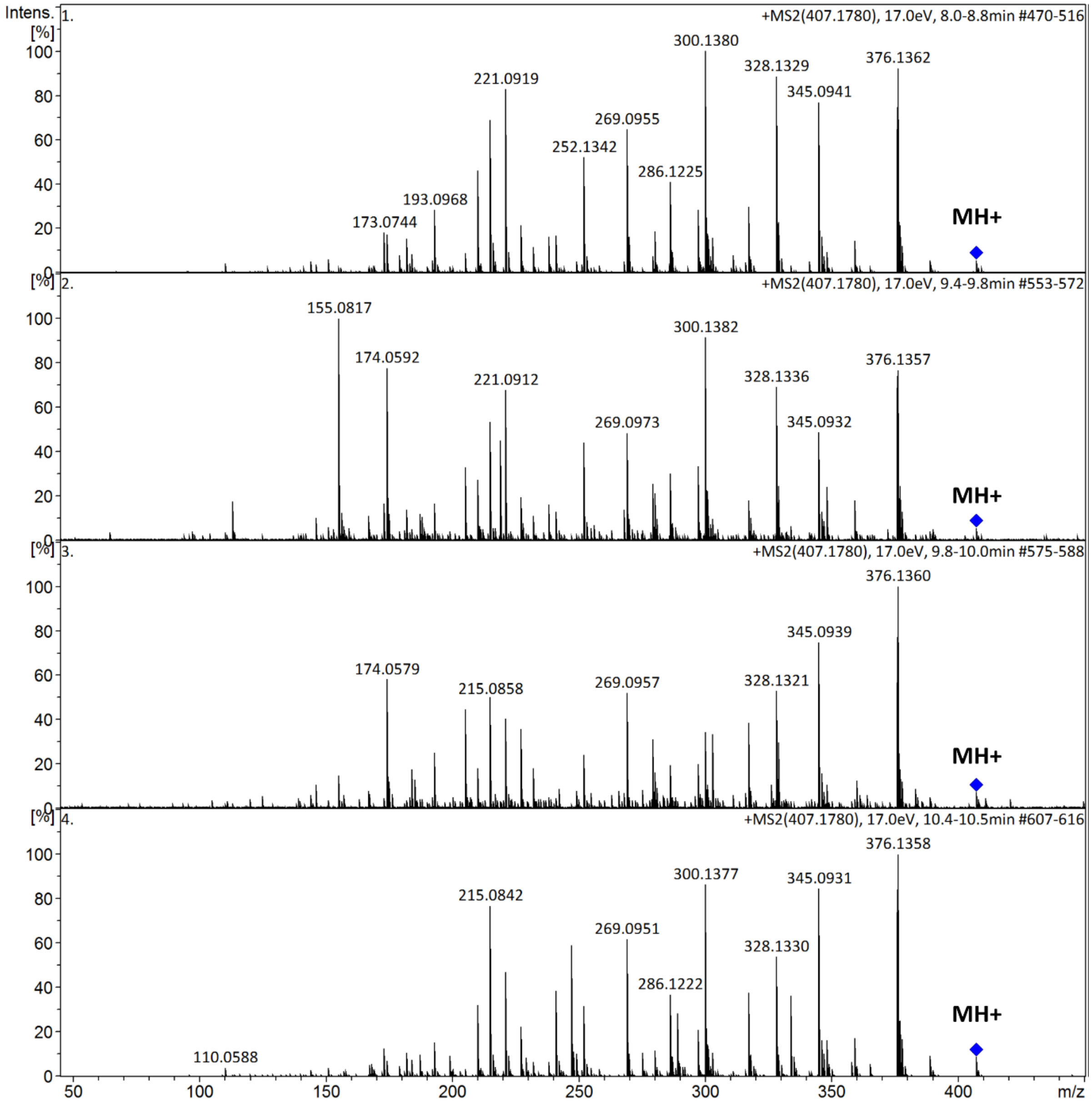

Figure S5 – panel A.

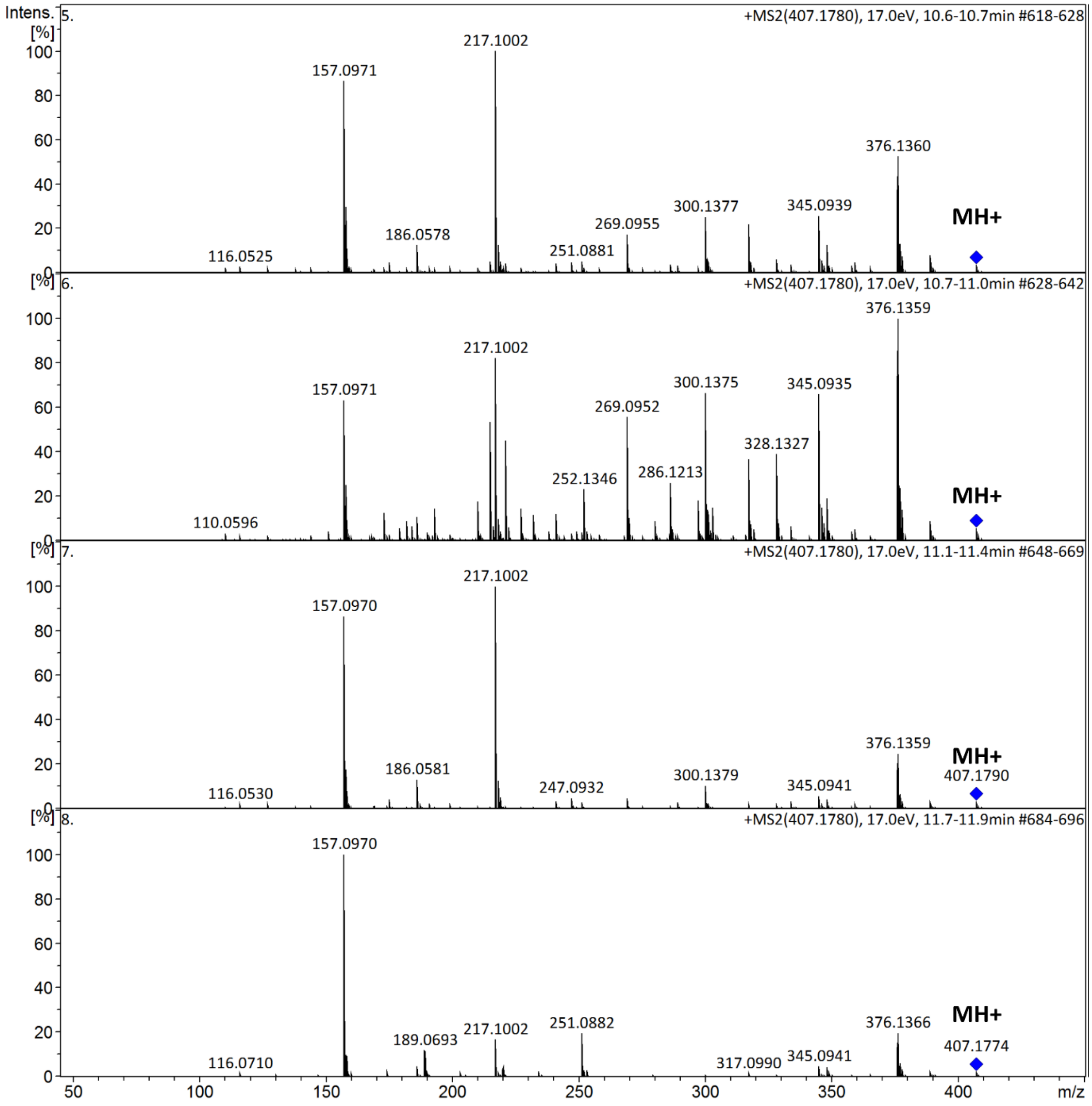

Figure S5 – panel B.

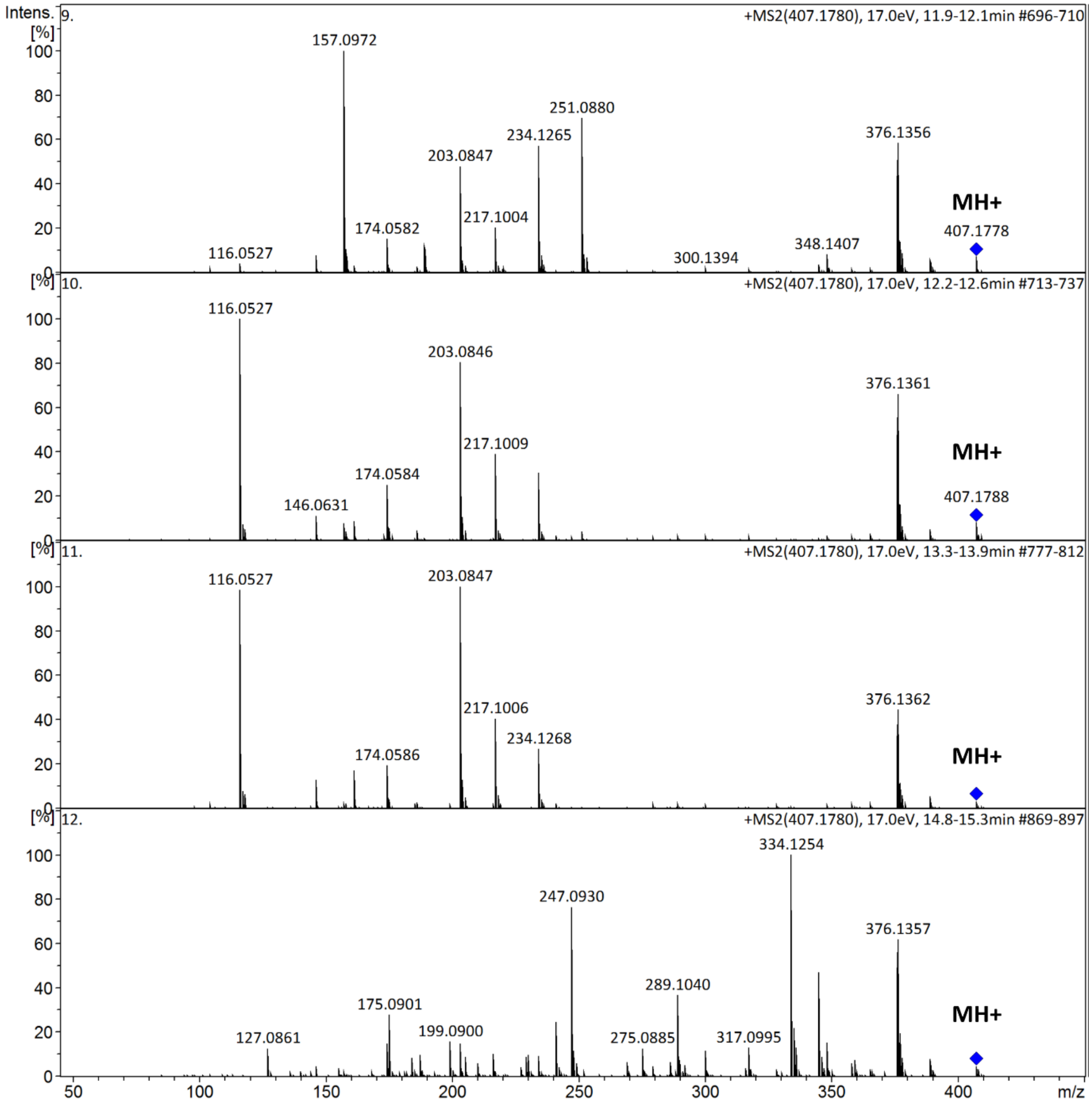

Figure S5 – panel C.

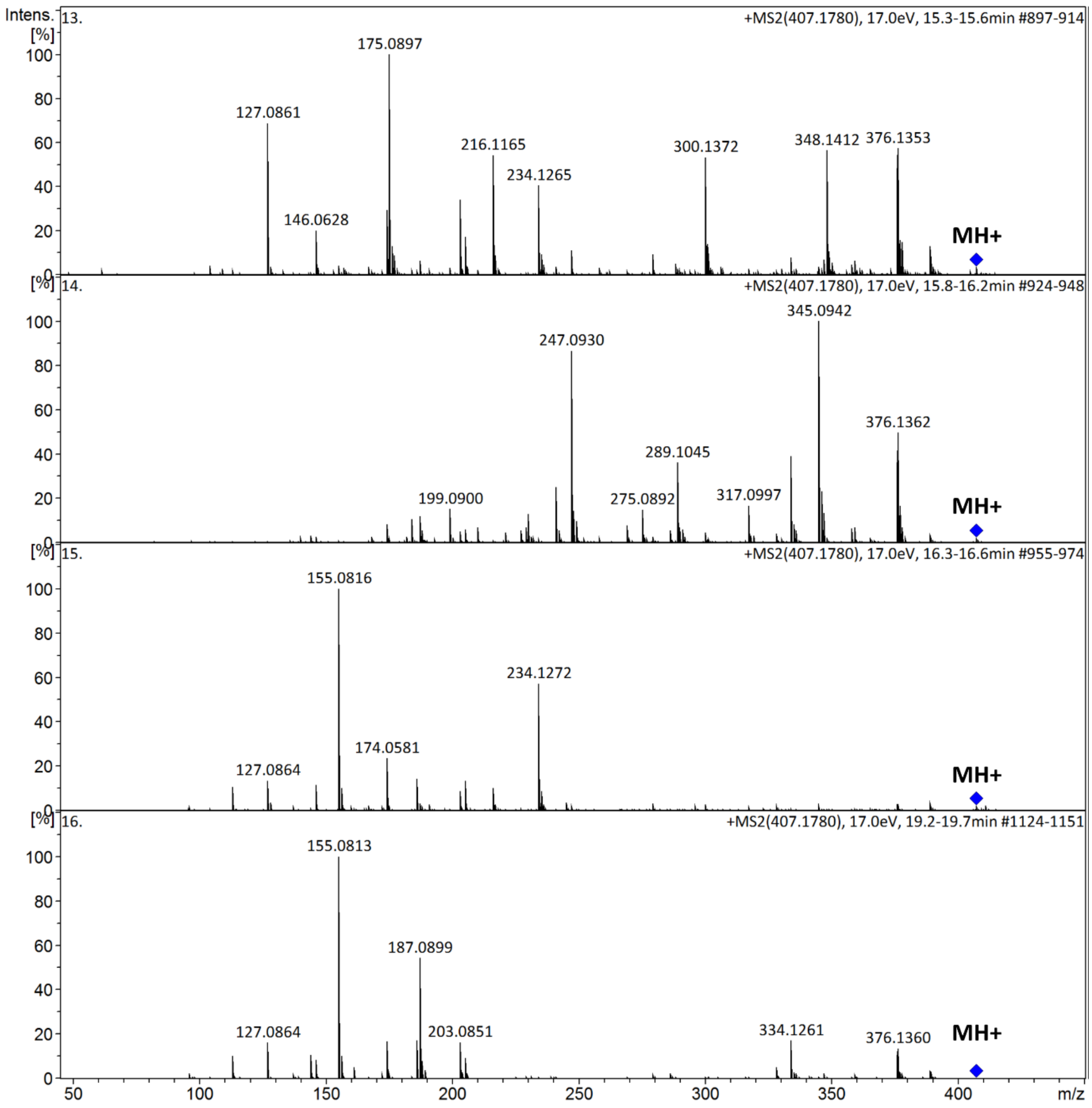

Figure S5 – panel D.

Figure S5. High-resolution MS/MS spectra of 16 products having *m/z* 407.1781 (see Table S1) correspond to a molecular weight MH^+ equivalent to two radicals (**αS**$^•$ and/or **αC**$^•$). The 16 spectra are shown in groups of 4 in the four panels **A**, **B**, **C** and **D**.

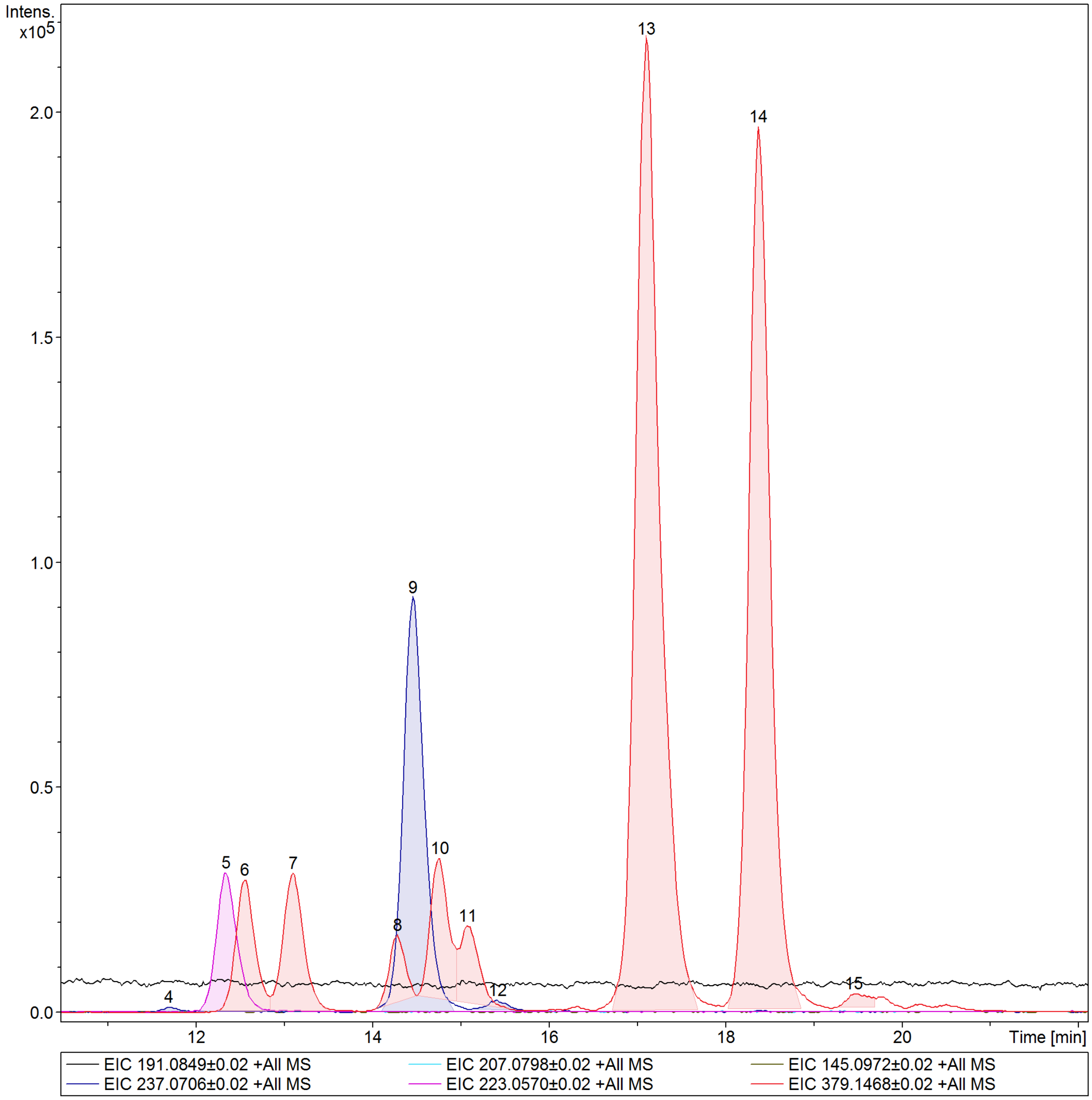

Figure S6. Fragments of LC-MS analysis for methylated Cys derivative **2** after irradiation at 800 Gy at pH 7.

Table S2. Conversion of Cys derivative **2** (peak 3) after irradiation at 800 Gy at pH 7. The peaks 4-15 are shown in Figure S5.

Peak	RT [min] [b]	Peak Intensity [c]	MH^+, *m*/*z*
1	1.7	335609	207.0798
2	1.8	17404	145.0972
3 [a]	4.4	2099126	191.0849
4	11.7	1153	237.0706
5	12.4	30942	223.0570
6	12.6	29417	379.1468
7	13.1	30908	379.1468
8	14.3	17094	379.1468
9	14.5	92186	237.0706
10	14.8	34178	379.1468
11	15.1	19134	379.1468
12	15.4	2605	237.0706
13	17.1	216303	379.1468
14	18.4	196846	379.1468
15	19.5	4144	379.1468

[a] Starting material (Cys derivative **2**); [b] retention time; [c] relative values.

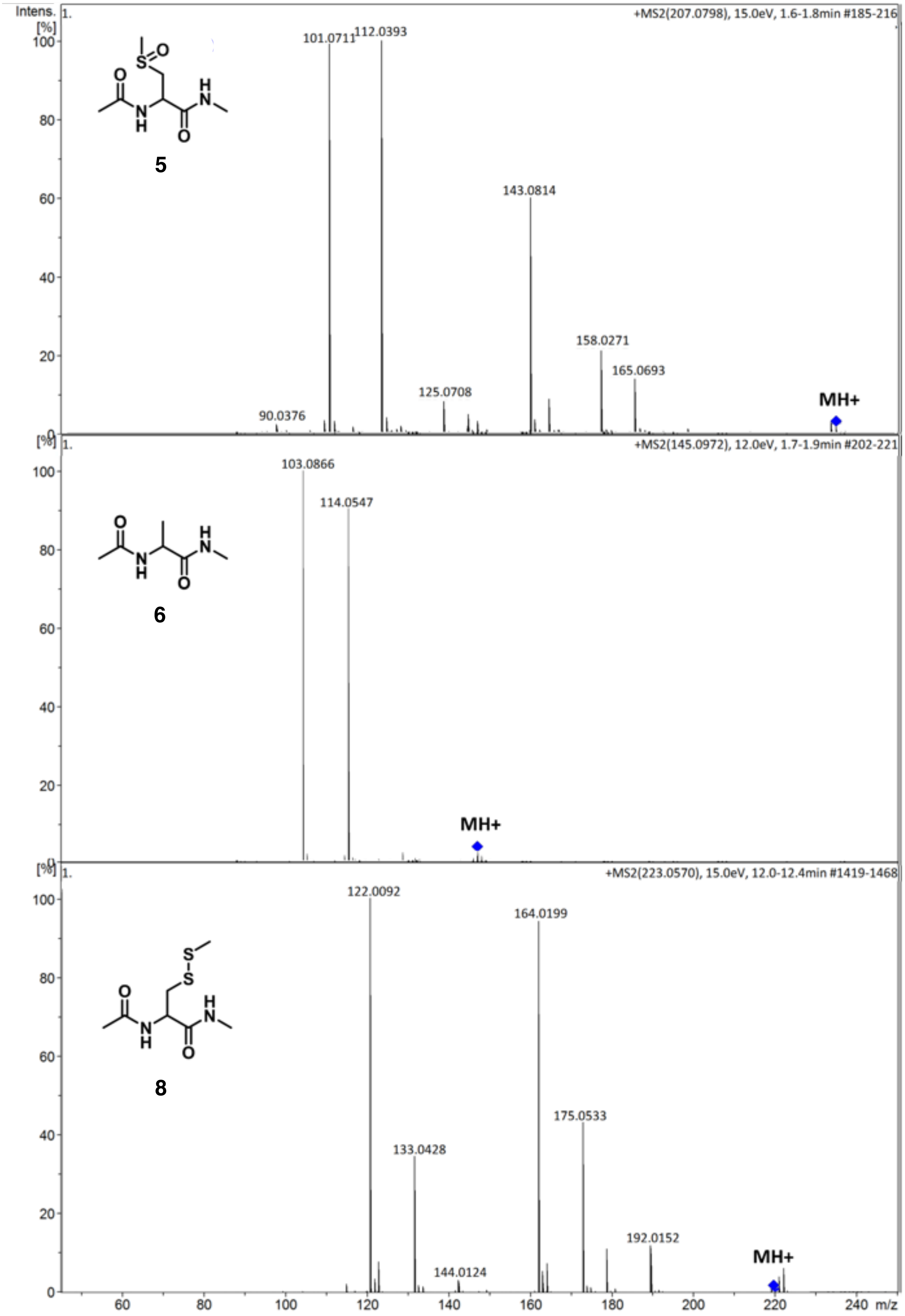

Figure S7. High-resolution MS/MS spectra of sulfoxide **5** (*m*/*z* 207.0798), alanine derivative **6** (*m*/*z* 145.0972) and disulfide **8** (*m*/*z* 223.0570).

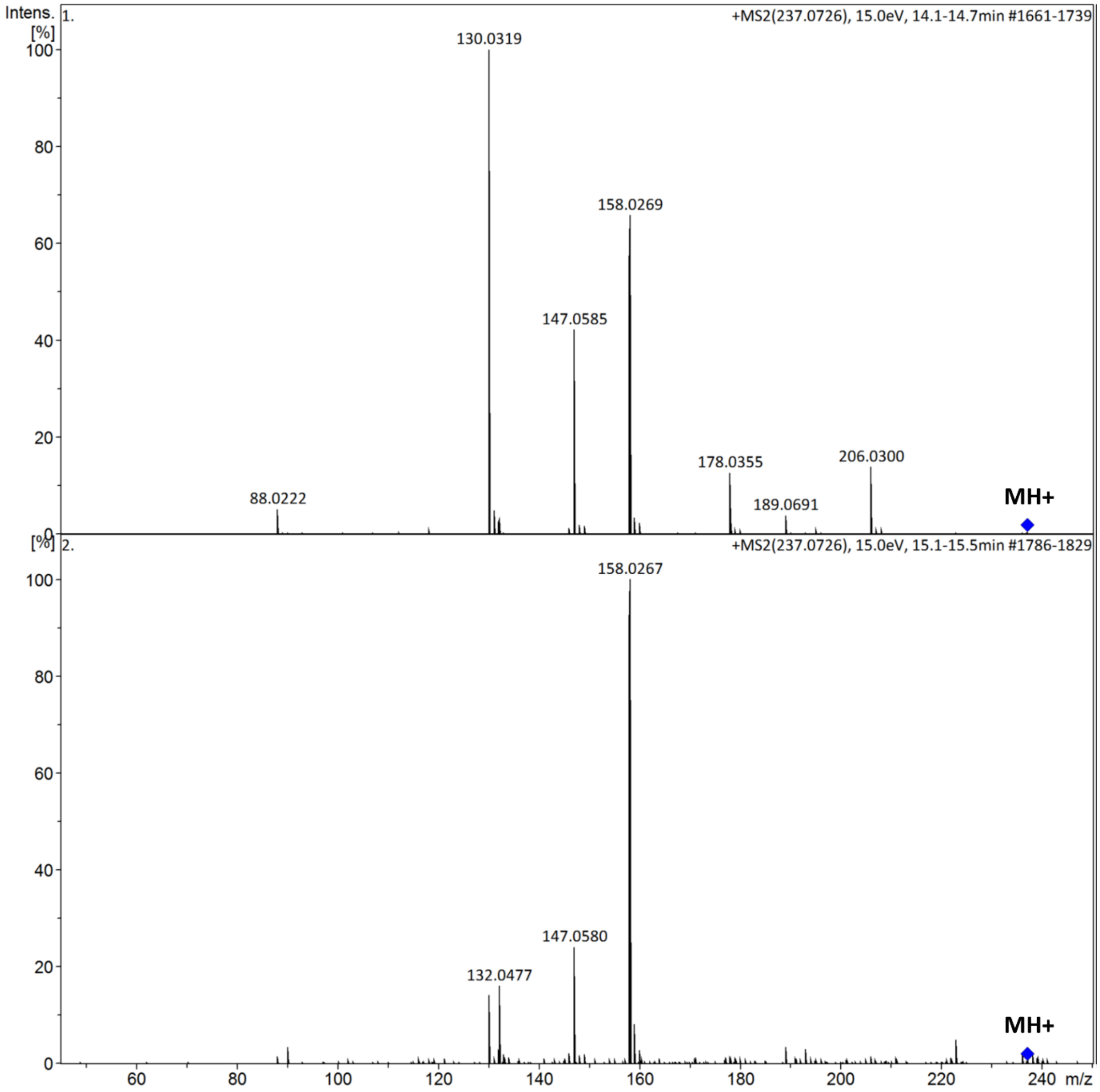

Figure S8. High-resolution MS/MS spectra of two of three products having *m/z* 237.0726; the third peak at RT 11.7 min have too low intensity to be registered (see Table S2). All of them are assigned to the cross-termination of **αC(2)•**, **αS(1)•** and **αS(2)•** with $CH_3S^•$, the most intense peak at RT 14.5 min is assigned to **αS(2)—**SCH_3 (Scheme S2).

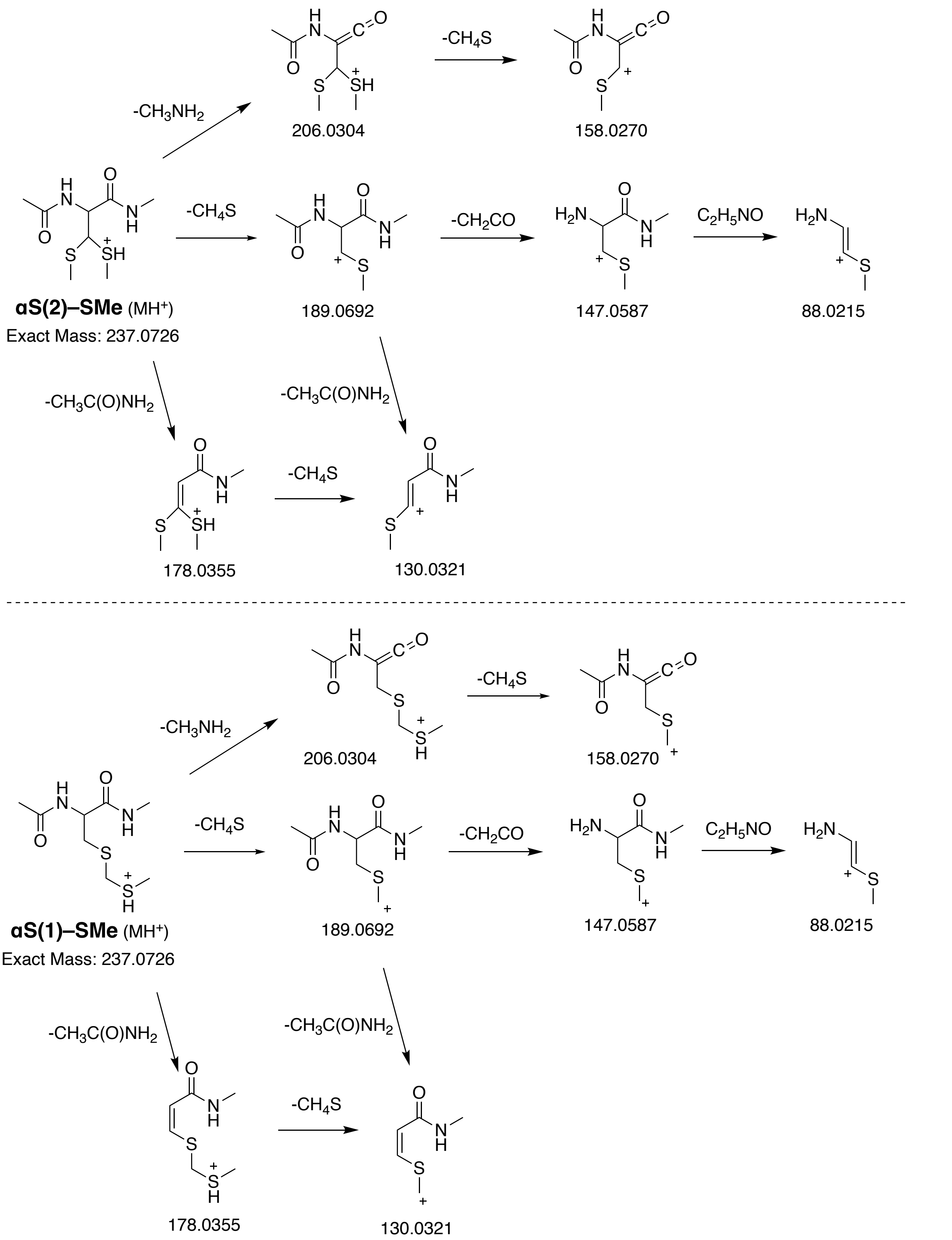

Scheme S2. Analysis of high-resolution MS/MS spectra of the main product at RT 14.5 min having *m/z* 237.0726 (see Figure S7 upper part) fits very well with both **αS(2)—**SCH_3 and **αS(1)—**SCH_3

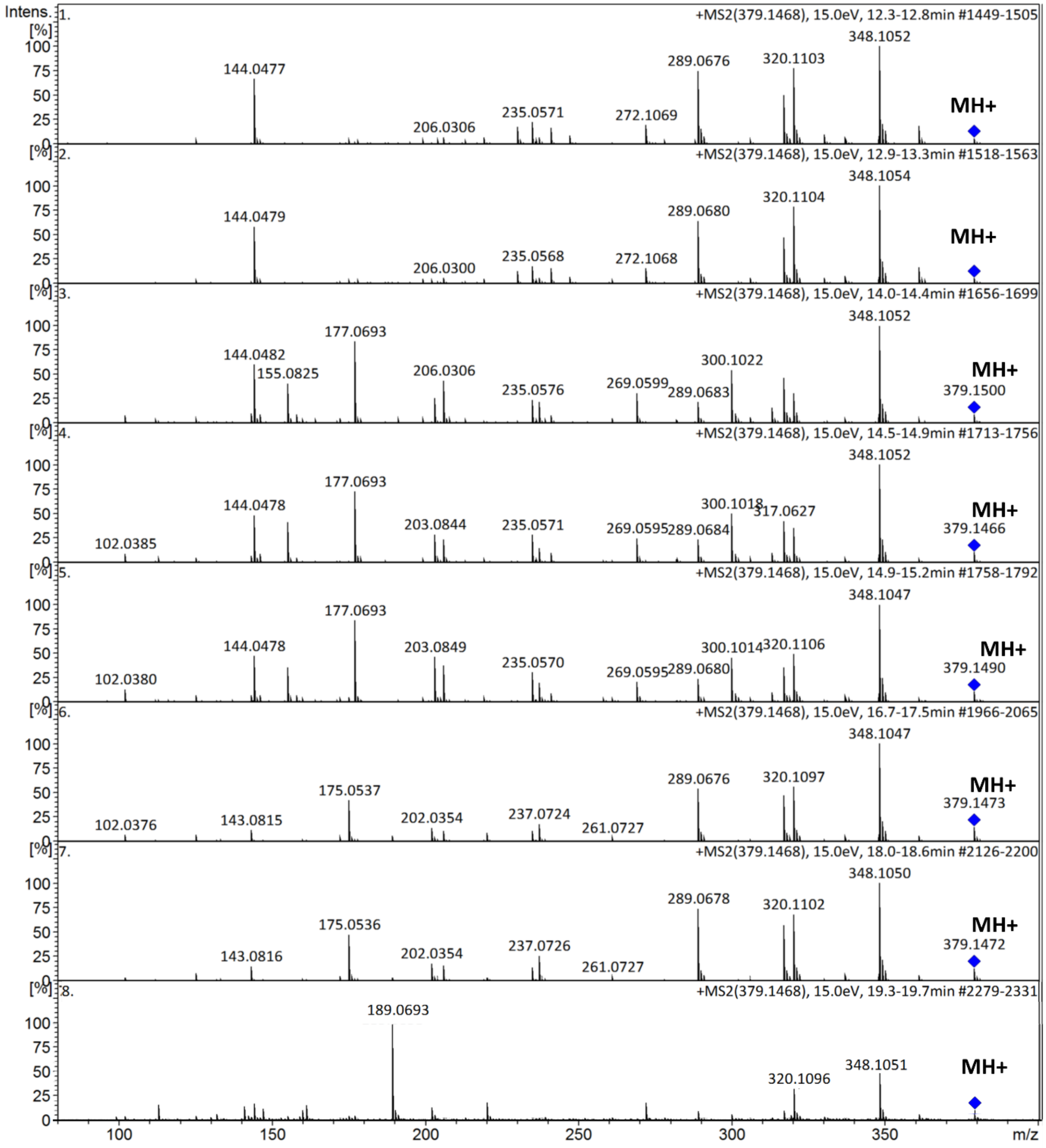

Figure S9. High-resolution MS/MS spectra of 8 products having *m/z* 379.1468 (see Table S1) corresponding to a molecular weight MH^+ equivalent to two radicals (**αS$^\bullet$** and/or **αC$^\bullet$**).
